# The prognostic value of miR-363-5p in colorectal cancer and its associated molecular mechanisms

**DOI:** 10.1186/s41065-026-00716-4

**Published:** 2026-08-03

**Authors:** Feng Xue, Yuanguo Si, Zhuoli Gao, Peng Hou

**Affiliations:** 1 Department of General Surgery III, Qingdao Traditional Chinese Medicine Hospital, Qingdao Hiser Hospital Affiliated of Qingdao University, Qingdao, Shandong 266033 China; 2 Department of Clinical Laboratory, Qingdao Traditional Chinese Medicine Hospital, Qingdao Hiser Hospital Affiliated of Qingdao University, No.4, Renmin Road, Shibei District, Qingdao, Shandong 266033 China; 3 Department of Cardiovascular Medicine I, Qingdao Traditional Chinese Medicine Hospital, Qingdao Hiser Hospital Affiliated of Qingdao University, No.4, Renmin Road, Shibei District, Qingdao, Shandong 266033 China

**Keywords:** Colorectal cancer, miR-363-5p, MEIS3, Prognosis

## Abstract

**Background:**

Colorectal cancer (CRC) is a prevalent malignant neoplasm characterized by high incidence and mortality rates. Currently, the role of microRNAs (miRNAs) in CRC is increasingly recognized. In this study, we aim to investigate the prognostic value of miR-363-5p and its associated molecular mechanisms.

**Methods:**

A total of 156 paired samples were collected from patients with CRC. The levels of miR-363-5p in the samples were detected by RT-qPCR. KM curves and multivariable Cox regression models evaluated the prognostic value of miR-363-5p. Flow cytometry, CCK-8 assay and Transwell assay examined the effects of miR-363-5p on cellular malignant phenotypes. Dual-luciferase reporter assays validated the targeting relationship.

**Results:**

The expression level of miR-363-5p was declined in CRC, and patients with TNM stage III + IV exhibited lower miR-363-5p expression compared to those with TNM stage I + II. Furthermore, individuals with low miR-363-5p expression demonstrated shorter overall survival than those with high expression, and miR-363-5p was confirmed as an independent protective factor for CRC. In vitro, miR-363-5p suppressed cellular malignant phenotypes, primarily manifested by miR-363-5p mimic promoting apoptosis while inhibiting proliferation, migration and invasion. Mechanistically, we identified MEIS3 as a direct target of miR-363-5p, and rescue experiments confirmed that MEIS3 overexpression attenuated the tumor-suppressive effects of miR-363-5p.

**Conclusions:**

Downregulated miR-363-5p were involved in CRC progression and associated with poor patient prognosis. Mechanistically, upregulated miR-363-5p suppressed the malignant cellular phenotype by negatively regulating MEIS3, thereby inhibiting the progression of CRC.

**Supplementary Information:**

The online version contains supplementary material available at https://doi.org/10.1186/s41065-026-00716-4.

## Background

Colorectal cancer (CRC) is a major cause of cancer-related morbidity and mortality worldwide [1]. Its risk factors are associated with multiple lifestyle elements, such as obesity, alcohol consumption and intake of processed meats [2]. During the period from 1990 to 2021, CRC-related deaths increased by approximately 83%, and China ranks among the countries with the highest incidence rates of CRC [3]. For an extended period, the treatment of CRC has predominantly relied on modalities such as surgery, chemotherapy, and radiotherapy [4]. For individuals with early-stage CRC, surgical resection remains the most effective treatment option. However, as the disease progresses, the efficacy of surgical intervention becomes limited. Chemotherapy and radiotherapy, as adjuvant therapies, primarily aim to reduce the risk of recurrence and improve survival rates [5]. Despite considerable progress in the treatment protocols for CRC, the prognosis of patients remains unfavorable. For CRC patients with distant metastases, the 5-year relative survival rate is only 14% [6].

The role of non-coding RNAs in CRC has recently gained increasing prominence. Numerous microRNAs (miRNAs) have been implicated in CRC risk and drug resistance [7–9]. They could influence tumor progression [10]. For example, miR-1204 promoted CRC cell proliferation and migration by directly targeting MASPIN, and its high expression was significantly associated with poorer patient survival rates [11]. Apart from non-coding RNAs, certain proteins and natural compounds have also been shown to modulate CRC [12, 13]. Additionally, miRNAs can act as sponges for non-coding RNAs, jointly influencing the proliferation and migration of CRC [14]. A previous study has shown that circ_0005939 exerted its pro-cancer effects by sponging miR-4693-3p, thereby upregulating UHRF1BP1L expression [15]. Critically, multiple miRNAs, including miR-1303 and miR-767-5p, demonstrated potential as prognostic biomarkers for CRC [16, 17]. Therefore, monitoring the levels of these miRNAs can help assess postoperative disease progression in patients, enabling faster and more accurate interventions for those requiring timely treatment.

A study has indicated that downregulated miR-363 were associated with carcinogenesis and metastasis [18]. The miR-363 precursor can generate two mature chains, miR-363-3p and miR-363-5p. Growing evidence suggested that miR-363-3p exerted an anti-cancer effect in CRC [19, 20]. For example, miR-363-3p inhibited the clonal survival, proliferation, and migration of CRC cells by directly targeting IFITM1 [20]. Furthermore, miR-363-3p was upregulated by Palmatine, thereby exerting a suppressive effect on CRC [21]. Notably, the levels of miR-363-5p was diminished in nasopharyngeal carcinoma (NPC), renal cell carcinoma (RCC) and hepatocellular carcinoma (HCC) [18, 22, 23]. In oral squamous cell carcinoma (OSCC), miR-363-5p was suppressed by the lncRNA MCM3AP-AS1, thereby promoting the proliferation, migration, and invasion of OSCC cells [24]. Nevertheless, the biological function of miR-363-5p in CRC remains poorly understood. Hence, we focused on miR-363-5p and aimed to (1) assess the expression and prognostic significance of miR-363-5p in CRC patients; (2) investigate the effects of miR-363-5p on CRC cell proliferation, apoptosis, migration, and invasion; and (3) elucidate whether MEIS3 serves as a direct functional target of miR-363-5p in CRC progression.

## Methods

### Study subjects

This research collected 156 paired samples from CRC participants, comprising CRC tissue and adjacent non-cancerous tissue. Tissue samples were stored at -80 °C. None of the patients had received radiotherapy, chemotherapy, or other antitumor treatments prior to surgery. Exclusion criteria: (1) Patients with concurrent malignant tumors; (2) Patients with major medical conditions or immunodeficiency diseases; (3) Pregnant or lactating women. Each participant underwent a 5-year postoperative follow-up.

The Ethics Committee of Qingdao Traditional Chinese Medicine Hospital, Qingdao Hiser Hospital Affiliated of Qingdao University has approved this investigation. Each participant has signed an informed consent form.

### Cell culture and processing

Human colon epithelial cells FHC were obtained from Yingwan Biotechnology and cultured in FHC complete cell culture medium (Yingwan). LoVo, SW480, HCT116 and SW620 were procured from SUNCELL and cultured in RPMI-1640 medium (ThermoFisher) supplemented with 1% penicillin-streptomycin (YEASEN) and 10% FBS (ThermoFisher). All cell lines were authenticated by short tandem repeat (STR) profiling performed immediately prior to the start of the experiments (within one month before use) and tested negative for mycoplasma contamination using a PCR-based assay. The STR profiles matched the reference standards with 100% concordance at all 8 core loci.

miR-363-5p mimic and negative control (mimic NC) were obtained from RiboBio. The pcDNA3.1 plasmid was purchased from ThermoFisher. miR-363-5p mimic and pcDNA3.1-MEIS3 (oe-MEIS3) were employed to overexpress miR-363-5p and MEIS3 in cells. According to the manufacturer’s instructions, they were transfected into SW480 and HCT116 cells using Lipofectamine 3000 Reagent (ThermoFisher). The MEIS3 siRNA (si-MEIS3) was synthesized by RiboBio. The sense sequence of si-MEIS3 was 5’-GCAAGGGAAAGAUGCCCAUC-3’, and the antisense sequence was 5’-GAUGGGCAUCUUUCCCUUGC-3’. Transfection was performed using Lipofectamine^®^ RNAiMAX Transfection Reagent (ThermoFisher) according to the manufacturer’s instructions. The final concentration of siRNA was 10 nM. The knockdown efficiency was evaluated by RT-qPCR at 48 h post-transfection. Moreover, after 48 h of transfection, cells were harvested for subsequent experiments.

### RT-qPCR

Tissues and cells were lysed with Trizol reagent (ThermoFisher) to extract total RNA. Subsequently, RNA was reverse transcribed into cDNA with the PrimeScript RT Reagent Kit (TaKaRa). Genes were amplified using TB Green Premix Ex Taq II FAST qPCR (TaKaRa) with cDNA as a template. Based on the results of the preliminary experiments (Ct value variation < 0.5 cycles across samples), we selected U6 as the internal control for miR-363-5p expression and GAPDH as the internal control for MEIS3 expression. Relative expression levels were calculated using the 2^−ΔΔCt^ method.

### Western Blot (WB)

Cells were lysed using RIPA Lysis buffer (Beyotime). Protein concentrations were measured with a BCA Protein Assay kit (ThermoFisher). The proteins were separated by SDS-PAGE, transferred to PVDF membranes (Millipore), and probed with primary antibodies against MEIS3 and GAPDH (Abcam), followed by HRP-conjugated secondary antibody. Signals were detected using SuperSignal West Pico PLUS (ThermoFisher).

### Dual-luciferase reporter assay

The binding sites of miR-363-5p and MEIS3 were identified using Targetscan database. Subsequently, the nucleic acid sequences of the MEIS3 3’ UTR, including wild-type (WT) and mutant (MUT) variants, were amplified in vitro via PCR and inserted into the pmirGLO vector (Promega). Using Lipofectamine 3000 Reagent, miR-363-5p mimic was co-transfected with either WT-MEIS3 or MUT-MEIS3 into SW480 and HCT116 cells. After 48 h, luciferase activity was measured using the Dual Luciferase Reporter Gene Assay Kit (YEASEN).

### Apoptosis detection

Apoptosis was detected using the Annexin V-FITC Kit (BD Biosciences). This method distinguishes early apoptotic cells (Annexin V+/PI-), late apoptotic cells (Annexin V+/PI+), and necrotic cells (Annexin V-/PI+). The total apoptosis rate was calculated as the sum of early and late apoptotic cells. Briefly, after washing collected cells twice with PBS, we prepared a cell suspension containing 5 × 10⁵ cells in 200 µL binding buffer. Then, 5 µL of FITC Annexin V and 1 µL of propidium iodide solution were added. Ultimately, apoptosis was determined by flow cytometry.

### Cell proliferation assay

Cells were seeded in a 96-well plate. Subsequently, cell proliferation was determined every 24 h using the Cell Counting Kit-8 (YEASEN). 10 µL of CCK-8 reagent was added to each well and incubated for 2 h.

### Migration and invasion detection

For migration assays, cells were first diluted to 1.5 × 10⁵ cells/well in RPMI-1640 medium devoid of FBS, and 200 µL was transferred to the Transwell chamber of the Transwell device (Corning). Complete medium was added to the lower chamber. After 48 h, the cell suspension in the Transwell chamber was removed, and the chamber was exposed to 4% paraformaldehyde for 20 min. Subsequently, cells were stained with crystal violet for 20 min. After staining, the inner wall cells were scraped off using a cotton swab. The Transwell chamber was then placed in a ventilated area to air-dry naturally for 2 h. For each well, five random fields were counted, and the assays were performed in three independent replicates. Data are presented as mean ± SD.

For invasion assays, melted the matrix gel (Corning) overnight at 4 °C and mixed it with RPMI-1640 medium without FBS. Next, dispensed 100 µL of diluted matrix gel onto the bottom of the Transwell chamber and incubated overnight. The following day, aspirated any residual fluid from the chamber. Then repeated the steps for the migration assay.

### Statistical analysis

Data were processed using IBM SPSS Statistics 27 and GraphPad Prism 9.3.1 and displayed as mean ± SD. For clinical samples, normality was assessed using the Shapiro-Wilk test, and homogeneity of variances was assessed using Bartlett’s test. In cell experiments, since each group contained only three biological replicates, we assumed that the data followed a normal distribution. Furthermore, homogeneity of variances was evaluated using the Brown-Forsythe test. Data met the assumptions of normality (Shapiro-Wilk test, *p* > 0.05) and homogeneity of variances (Bartlett’s test, *p* > 0.05; Brown-Forsythe test, *p* > 0.05). Therefore, differential expression of miR-363-5p and MEIS3 was analyzed by paired t-tests. Comparisons among three or more groups were performed using one-way or two-way ANOVA followed by Tukey’s post hoc test. Collinearity diagnostics were performed using variance inflation factor (VIF). The goodness-of-fit of the logistic regression model was assessed using the Hosmer-Lemeshow test. KM curves and multivariable Cox regression analysis demonstrated the prognostic value of miR-363-5p. The correlation between miR-363-5p and MEIS3 was analyzed by Pearson correlation test using CRC tumor tissues (*n* = 156). *p* < 0.05 was considered statistically significant.

## Results

### Low levels of miR-363-5p were involved in the progression of CRC and associated with poor patient prognosis

**Fig. 1:**
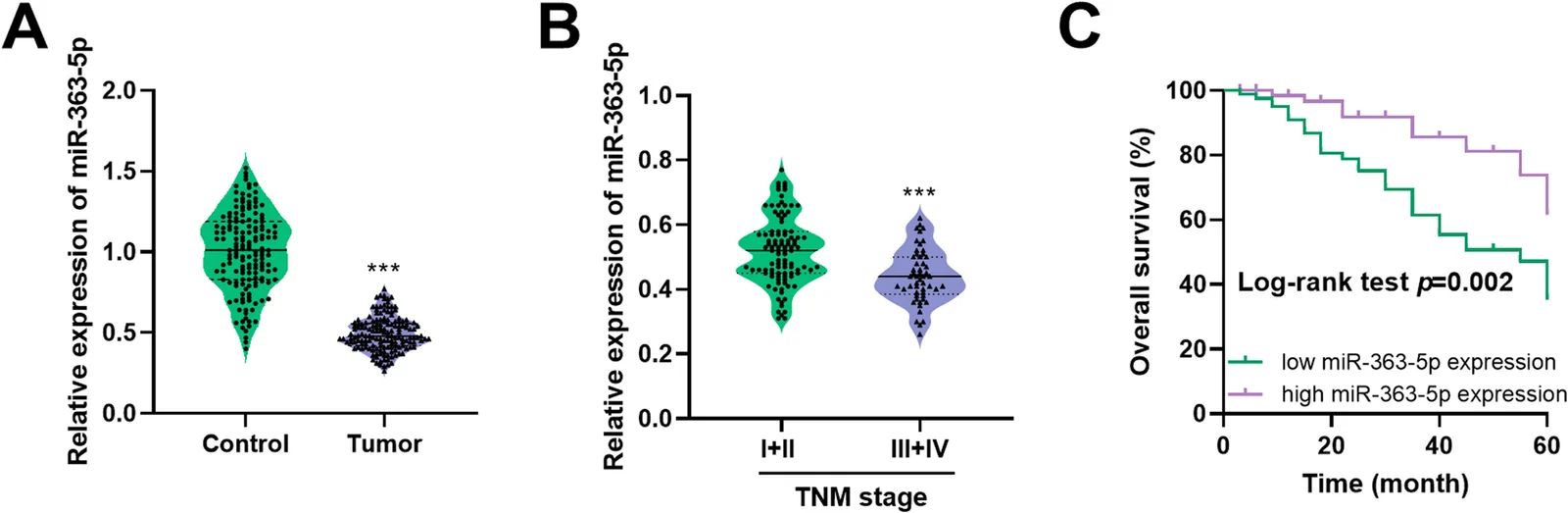
The levels of miR-363-5p in CRC. Data are shown as mean ± SD. ***p < 0.001. **A** The results of the paired t-test indicated that levels of miR-363-5p were reduced in CRC tissues (*n* = 156) compared with the control group (*n* = 156). **B** The results of the unpaired t-test showed that miR-363-5p levels were significantly lower in patients with TNM stage III + IV (*n* = 53) than in those with TNM stage I + II (*n* = 103). **C** The overall survival period was shorter in the low miR-363-5p expression group than in the high expression group

To determine the clinical significance of miR-363-5p in CRC, we first examined its expression levels in clinical samples. Tumor tissue and adjacent non-cancerous tissue were gathered from 156 CRC patients. Paired t-tests uncovered that the content of miR-363-5p was diminished in CRC tissues (Fig. 1A). Subsequently, patients were stratified according to TNM staging. Compared with TNM I + II stage individuals, miR-363-5p levels were lower in TNM III + IV stage individuals (Fig. 1B).

Based on the mean relative expression of miR-363-5p (0.49), individuals were divided into a low-expression group (mean expression < 0.49, *n* = 83) and a high-expression group (mean expression ≥ 0.49, *n* = 73). Chi-square test results indicated significant differences between the two groups in TNM stage and tumor differentiation (Table 1). Additionally, univariate and multivariate logistic regression analyses further confirmed that low miR-363-5p expression was independently associated with advanced TNM stage and poor tumor differentiation (Table 2). KM curve demonstrated that the overall survival of participants in the low-expression group was inferior to that of the high-expression group (Fig. 1C). Multivariable Cox regression analysis indicated that high miR-363-5p expression and high tumor differentiation were independent protective prognostic factor for CRC patients, while larger tumor size and advanced TNM stage were independent risk factors (Table 3).

**Table 1:** The relationship between miR-363-5p expression in CRC patients and clinical indicators

Parameters	Patients (*n* = 156)	The expression of miR-363-5p		*p*
		Low expression (*n* = 83)	High expression (*n* = 73)	
Age (years)				0.501
< 60	75	42	33	
≥ 60	81	41	40	
Gender				0.514
Male	77	43	34	
Female	79	40	39	
Tumor size (cm)				0.166
< 4	87	42	45	
≥ 4	69	41	28	
TNM stage				0.003**
I-II	103	46	57	
III-IV	53	37	16	
Tumor differentiation				0.022*
Poor	94	57	37	
Moderate/high	62	26	36	

**p < 0.01, *p < 0.05

**Table 2:** Univariate and multivariate logistic regression analysis of factors associated with miR-363-5p expression in CRC patients

Parameters	Univariate analysis		Multivariate analysis	
	OR (95%CI)	*p*	OR (95%CI)	*p*
Age	1.242 (0.661–2.333)	0.501		
Gender	0.811 (0.432–1.523)	0.514		
Tumor size	0.637 (0.337–1.207)	0.167		
TNM stage	0.349 (0.173–0.705)	0.003**	0.069 (0.019–0.248)	< 0.001
Tumor differentiation	0.469 (0.244-0.900)	0.023*	0.089 (0.026–0.309)	< 0.001

Categorical variables were coded as follows: TNM stage (0 = I-II, 1 = III-IV); tumor differentiation (0 = moderate/high, 1 = poor)

**p < 0.01, * *p* < 0.05

**Table 3:** Multivariable Cox regression analysis of prognostic parameters in patients with CRC

Parameters	HR	95% CI for HR		*p*
		Lower	Upper	
miR-363-5p	0.219	0.084	0.572	0.002**
Age	1.929	0.236	15.739	0.540
Gender	1.603	0.177	14.492	0.675
Tumor size	2.810	1.017	7.762	0.046*
TNM stage	6.038	1.470	24.796	0.013*
Tumor differentiation	0.354	0.141	0.885	0.026*

Categorical variables were coded as follows: TNM stage (0 = I-II, 1 = III-IV); tumor differentiation (0 = poor, 1 = moderate/high)

**p < 0.01, * *p* < 0.05

### miR-363-5p mimic suppressed the malignant cellular phenotype

**Fig. 2:**
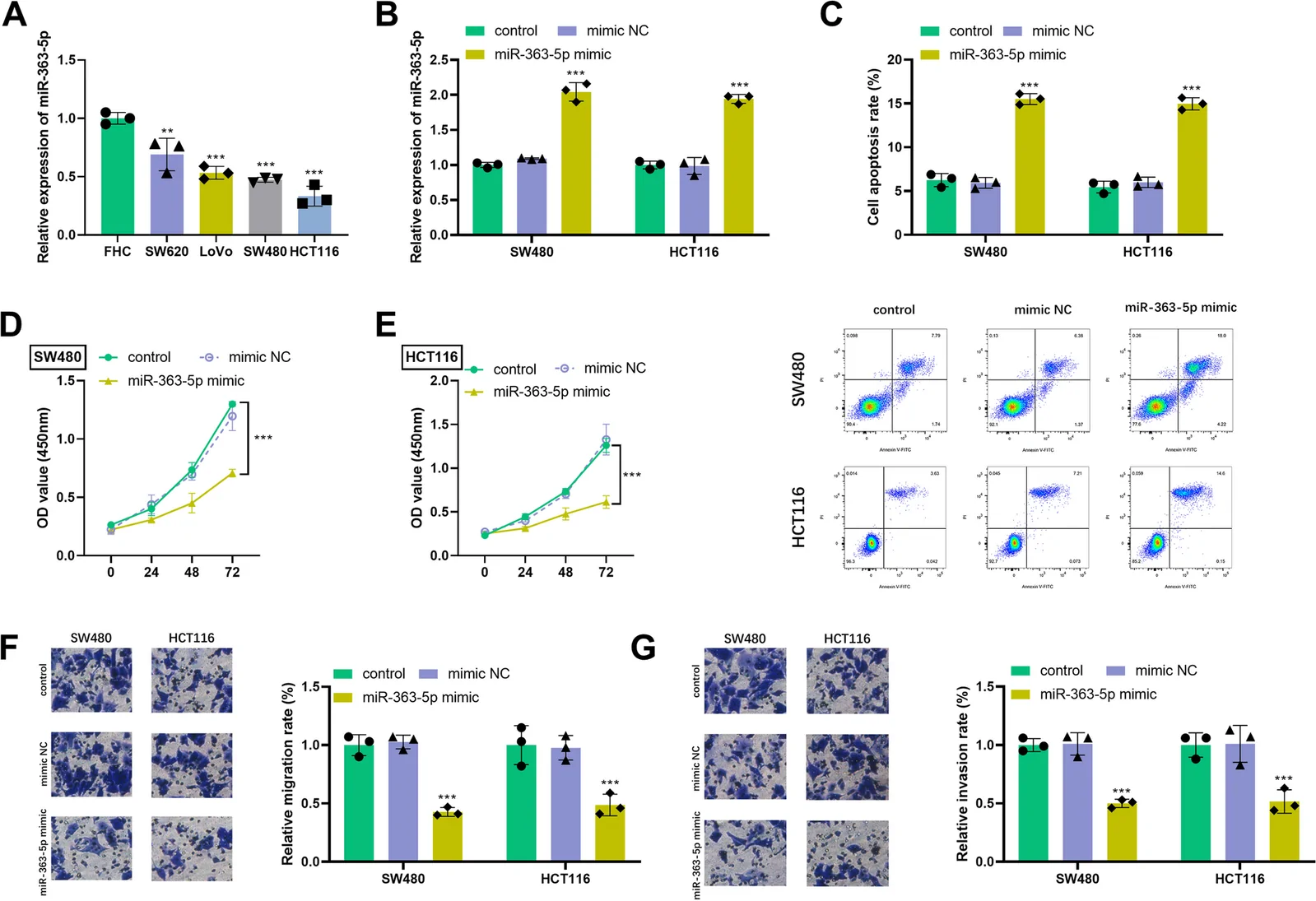
miR-363-5p modulate the malignant phenotype of CRC cells. Data are shown as mean ± SD. Each group consisted of three biological replicates. ***p < 0.001, **p < 0.01. **A** The content of miR-363-5p was significantly reduced in CRC cell lines. **B** miR-363-5p mimic promoted the levels of miR-363-5p in cells. **C** Upregulated miR-363-5p promoted apoptosis. **D–G** miR-363-5p upregulation depressed proliferation, migration and invasion. Cell proliferation analysis at 48 h and 72 h were performed using two-way ANOVA with Tukey’s post-hoc test. Other experiments were analyzed using one-way ANOVA with Tukey’s post-hoc test. The migration and invasion images shown in Figs. 2F–G are representative of three independent experiments with similar results

Having established the clinical relevance of miR-363-5p, we next investigated its functional effects on CRC cells. One-way ANOVA compared differences in miR-363-5p levels across various CRC cell lines. The findings revealed varying degrees of miR-363-5p downregulation, with the most pronounced decline observed in SW480 and HCT116 cells (Fig. 2A). Therefore, we transfected miR-363-5p mimic into these two cell lines and found it remarkably enhanced miR-363-5p expression (Fig. 2B). Moreover, upregulation of miR-363-5p significantly increased the percentage of both early apoptotic and late apoptotic cells compared with the control group, leading to a marked increase in total apoptosis rate (Fig. 2C). Furthermore, miR-363-5p mimic inhibited proliferation, migration, and invasion, thereby suppressing the malignant phenotype (Fig. 2D-G).

### A binding site existed between miR-363-5p and MEIS3

**Fig. 3:**
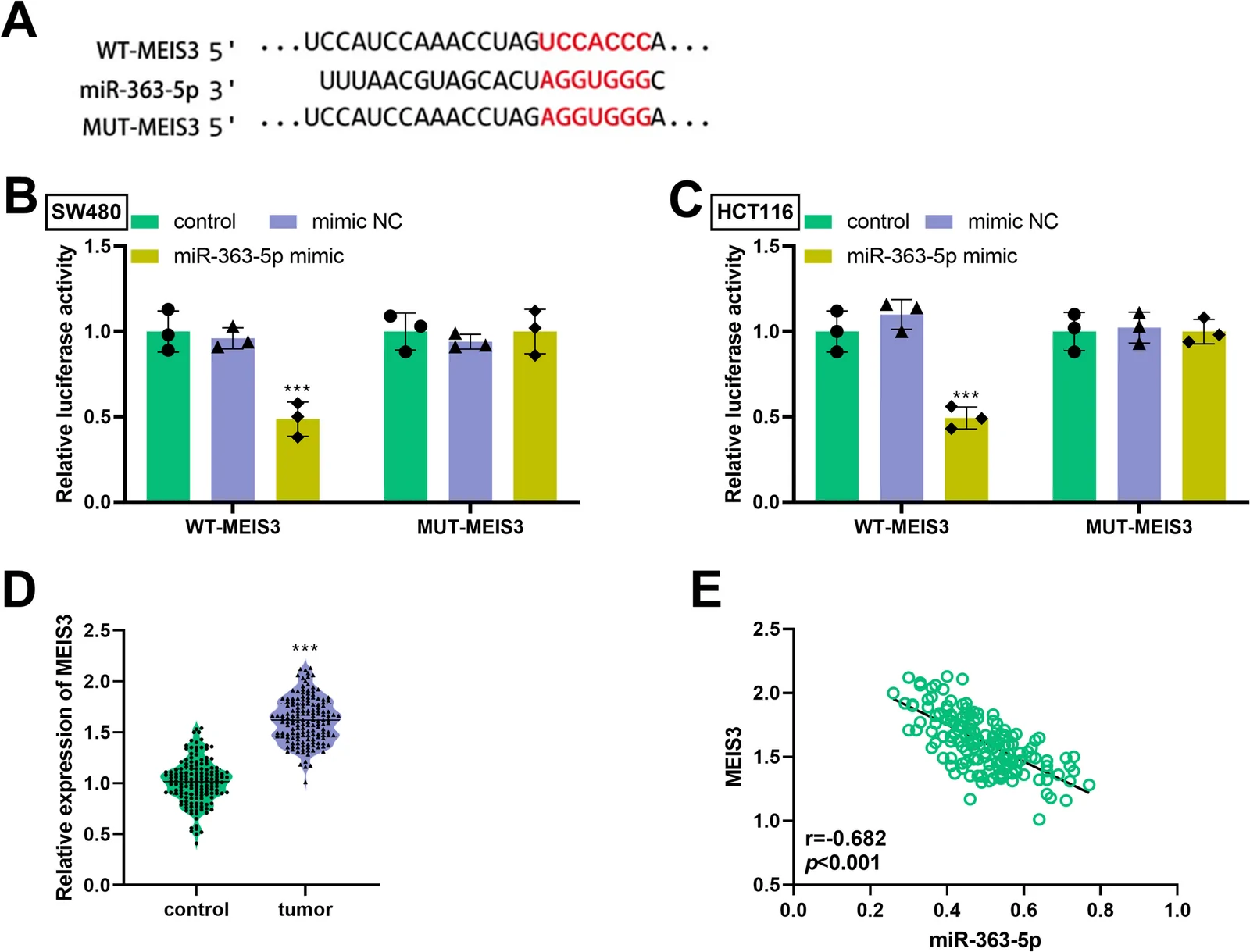
MEIS3 was a target site of miR-363-5p. Data are shown as mean ± SD. ***p < 0.001. **A** The binding site of miR-363-5p and MEIS3. **B-C** miR-363-5p mimic suppressed luciferase activity of WT-MEIS3. However, it had no significant effect on MUT-MEIS3 (*n* = 3 independent biological replicates). **D** The results of the paired t-test revealed that MEIS3 expression was elevated in CRC tissues (*n* = 156). **E** Pearson correlation analysis indicated that miR-363-5p negatively correlated with MEIS3

Utilizing the Targetscan database, a binding site between miR-363-5p and MEIS3 was identified (Fig. 3A). Additionally, the miR-363-5p mimic markedly suppressed luciferase activity in WT-MEIS3, whereas no significant difference was observed in MUT-MEIS3 (Fig. 3B-C). Paired t-tests revealed elevated MEIS3 levels in CRC tissues and there was a negative correlation between MEIS3 and miR-363-5p (Fig. 3D-E).

### miR-363-5p exerted tumor-suppressive effects by negatively modulating MEIS3

**Fig. 4:**
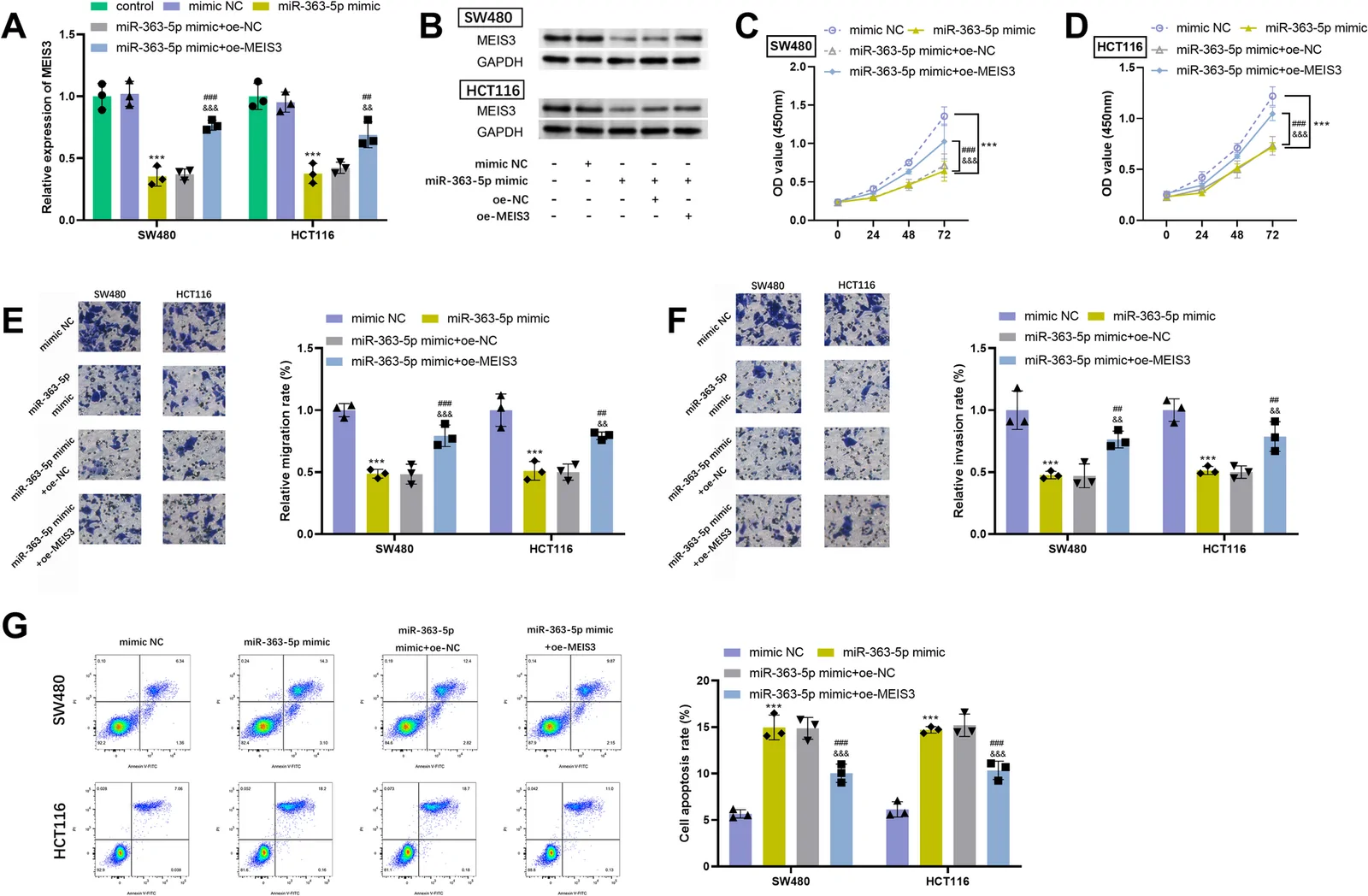
miR-363-5p suppressed the malignant phenotype of cells by regulating MEIS3. Data are shown as mean ± SD. Each group consisted of three biological replicates. ***p < 0.001, compared with the control group; ^###^*p* < 0.001, ^##^*p* < 0.01, compared with the miR-363-5p mimic group. ^&&&^*p* < 0.001, ^&&^*p* < 0.01, compared with the miR-363-5p mimic + oe-NC group. **A** miR-363-5p mimic suppressed MEIS3 expression in CRC cells. oe-MEIS3 partially restored its levels. **B** The miR-363-5p mimic reduced MEIS3 protein levels, which were reversed by overexpression of MEIS3. **C–F** oe-MEIS3 reversed the suppression of proliferation, migration, and invasion caused by upregulated miR-363-5p. **G** Overexpression of MEIS3 mitigated the pro-apoptotic effect of the miR-363-5p mimic. Cell proliferation analysis at 72 h were performed using two-way ANOVA with Tukey’s post-hoc test. Other experiments were analyzed using one-way ANOVA with Tukey’s post-hoc test. The migration and invasion images shown in Figs. 4E–F are representative of three independent experiments with similar results

To validate whether MEIS3 mediates the tumor-suppressive effects of miR-363-5p, we performed rescue experiments by overexpressing MEIS3 in miR-363-5p mimic-transfected cells. In CRC cells, upregulated miR-363-5p suppressed MEIS3 levels, while oe-MEIS3 partially restored MEIS3 levels (Fig. 4A). The WB results showed a similar trend, indicating that transfection with the miR-363-5p mimic significantly reduced MEIS3 protein expression in both SW480 and HCT116 cells. However, oe-MEIS3 in the presence of the miR-363-5p mimic partially restored MEIS3 protein levels (Fig. 4B). Regarding cellular malignant phenotypes, MEIS3 overexpression mitigated the miR-363-5p mimic’s promotion of apoptosis and its depression of proliferation, migration and invasion (Fig. 4C-G).

Subsequently, we knocked down endogenous MEIS3 using siRNA in CRC cells. The knockdown efficiency of si-MEIS3 was evaluated by RT-qPCR at 48 h post-transfection. The results showed that si-MEIS3 reduced MEIS3 mRNA levels by approximately 48% in both SW480 and HCT116 cells compared with the control group (Supplementary Fig. 1A). Moreover, MEIS3 inhibition promoted apoptosis while suppressing proliferation, migration, and invasion (Supplementary Fig. 1B-F).

## Discussion

CRC ranks as the fourth most common cancer worldwide, accounting for approximately 10% of all cancer cases [25, 26]. Despite great advances in treatment, the 5-year overall survival rate for patients with metastatic disease remains unfavorable [26]. In oncology, miRNAs have garnered extensive attention due to their dual roles as oncogenic miRNAs (miR-21) or tumor-suppressing miRNAs (miR-16-5p) [27, 28]. They have been discovered to be associated with the cell cycle, immune evasion and chemotherapy resistance [29–31]. Furthermore, miRNAs demonstrate significant potential as prognostic biomarkers for CRC [16, 17]. This suggests that postoperative physical conditions of CRC patients can be monitored via miRNAs, which is conducive to timely and effective treatment and can enhance survival rates.

The levels of miR-363-5p were downregulated in multiple tumors, involving RCC and NPC [22, 23]. Plasma exosomal miR-363-5p exerted a tumor-suppressive role in breast cancer [32]. Furthermore, it was demonstrated that miR-363-5p influenced the level of vascular secretory factors and the angiogenic characteristics of endothelial cells [33]. The above cases revealed the potential for miR-363-5p to modulate CRC. We found a decline in miR-363-5p content within CRC, a pattern consistent with reports from other tumor types [22, 23]. Furthermore, individuals in the low miR-363-5p expression group exhibited shorter survival times. Multivariable Cox regression analysis demonstrated that miR-363-5p acted as an independent protective factor for CRC. Consequently, we conclude that miR-363-5p has potential as a prognostic biomarker for CRC.

miR-363-5p frequently functioned as an lncRNA sponge [24]. For example, miR-363-5p, acting as a sponge for the lncRNA PITPNA-AS1, participated in HCC progression by modulating PDGFD [18]. FOXD2-AS1 facilitated NPC progression via modulating the miR-363-5p/S100A1 signaling pathway [22]. Moreover, miR-363-5p can attenuate LPS-induced proliferation and inflammation in Schwann cells [34]. It has been uncovered that miR-363-5p suppressed CRC progression by promoting apoptosis and inhibiting proliferation, migration, and invasion.

MEIS3 is a transcription factor associated with carcinogenic pathways [35]. Previous studies have demonstrated its involvement in HCC progression and its potential impact on the HCC immune microenvironment [36, 37]. Additionally, MEIS inhibitor reduced the survival rate of primary leukemia cells and stem cells by inducing apoptosis [38]. Mechanistically, MEIS3 has been shown to enhanced CRC cell migration and invasion by regulating downstream effectors including LAMB1, MMP2, and vimentin [39]. Additionally, MEIS3 was identified as a cetuximab-sensitive gene in CRC, where it modifies drug sensitivity through the c-Met and Akt signaling pathways [40]. These findings suggest that MEIS3 not only facilitates cancer cell metastasis but also contributes to therapeutic resistance. Furthermore, a recent study demonstrated that MEIS3 nuclear translocation activated macrophage colony-stimulating factor expression, thereby promoting an immunosuppressive tumor microenvironment and contributing to anti-PD-1 resistance in CRC [41]. Collectively, these reports indicate that MEIS3 functions as a multifunctional transcription factor regulating downstream networks involved in metastasis, immune modulation, and drug resistance. We observed upregulation of MEIS3 in CRC patients which is consistent with a previous report [39]. More importantly, upregulation of MEIS3 alleviated the suppressive effect of miR-363-5p mimic on the malignant phenotype. Therefore, we speculated that low levels of miR-363-5p may facilitate CRC cell migration and invasion by negatively modulating MEIS3, thereby exerting its tumor-promoting role in CRC.

### Limitations and future directions

When interpreting the results, several limitations must be acknowledged. (1) The clinical sample size was limited and relatively homogeneous in origin. Future studies should expand both the sample size and diversity of sources. (2) Due to limited availability of clinical tissue samples, MEIS3 protein expression was not assessed by western blotting in our CRC tissues. However, a previous study has demonstrated that the expression of the MEIS3 protein was elevated in CRC [39]. In the future, we plan to conduct a study using an independent cohort to validate the correlation between miR-363-5p and MEIS3 at the protein level. (3) While this study explored miR-363-5p’s suppression of CRC progression via MEIS3, other target sites interacting with miR-363-5p may exist. Therefore, additional studies are warranted. (4) Prior research indicated that MEIS3 was associated with CRC drug resistance. Therefore, exploring the direct role of the miR-363-5p/MEIS3 axis in this context represents a key focus for future research. (5) The Transwell assays were conducted over a period of 48 h. This relatively long duration may allow cell proliferation to confound the results, as an increase in migrated or invaded cells could reflect enhanced proliferation rather than improved motility. To address this issue in future studies, we plan to employ parallel control experiments using a proliferation inhibitor (e.g., mitomycin C) to exclude this potential confounding effect.

## Conclusions

Our investigation demonstrated that the downregulation of miR-363-5p were involved in CRC progression and associated with poor patient prognosis. Mechanistically, the upregulation of miR-363-5p inhibited the malignant cellular phenotype by negatively regulating MEIS3, thereby repressing CRC progression. This study innovatively explores the regulatory mechanism of miR-363-5p in CRC. It not only provides a potential prognostic biomarker for CRC but also offers a promising therapeutic target.

## Supplementary Information

Supplementary Material 1: Supplementary Fig. 1 The effect of MEIS3 knockdown on cell phenotype. Data are shown as mean ± SD. Each group consisted of three biological replicates. ***p < 0.001. A si-MEIS3 significantly suppressed MEISE3 expression in CRC cells. B–F Transfection with si-MEIS3 significantly enhanced apoptosis while inhibiting proliferation, migration, and invasion. Cell proliferation analysis at 48 h and 72 h were performed using two-way ANOVA with Tukey’s post-hoc test. Other experiments were analyzed using one-way ANOVA with Tukey’s post-hoc test.

## Data Availability

The datasets generated during and/or analysed during the current study are available from the corresponding author on reasonable request.

## Abbreviations

CRC: Colorectal cancer
miRNAs: microRNAs
NPC: Nasopharyngeal carcinoma
RCC: Renal cell carcinoma
WT: Wild-type
